# Numerosity Comparison, Estimation and Proportion Estimation Abilities May Predict Numeracy and Cognitive Reflection in Adults

**DOI:** 10.3389/fnhum.2021.762344

**Published:** 2021-11-23

**Authors:** Midori Tokita, Sumire Hirota

**Affiliations:** ^1^Faculty of Health Sciences, Mejiro University, Saitama, Japan; ^2^Graduate School of Environmental and Information Studies, Tokyo City University, Yokohama, Japan

**Keywords:** numerosity comparison, numerosity estimation, proportion estimation, approximate number system, numeracy, cognitive reflection

## Abstract

This study explores whether and how different tasks associated with approximate number system (ANS) ability are related to numeracy and cognitive reflection in adults. We conducted an online experiment using a sample of 300 Japanese adults aged 20–39. Participants were given three ANS tasks (numerosity comparison, numerosity estimation, and proportion estimation) as well as Rasch-based numeracy scale and cognitive reflection test, and we tested the correlation among the measures of these tasks. We explored the hypothesis that the typical measures used to gauge ANS ability, numerosity comparison and numerosity estimation may mediate different cognitive mechanisms in adults. We also introduced a task measuring proportion estimation, added because such estimation requires numerosity perception and the ability to map symbolic numerals. Our findings suggest that there is a weak, but significant correlation among the three ANS-related tasks. Moreover, there is a significant relationship between each of these measures and the numeracy and CRT score, suggesting that the ANS-related ability may be associated with higher cognitive abilities such as numeracy and cognitive reflection. In addition, we found that performances on the numerosity and proportion estimation are more clearly related to CRT score than the numerosity comparison task.

## Introduction

The ability to extract the approximate numerical values of objects/events is crucial for surviving in the natural world as well as in modern society, which is full of numerical information in everyday life. Several studies on behavioral, neurophysiological, and brain imaging have demonstrated a dedicated mechanism for this numerical ability, denoted as the approximate number system (ANS), in humans as well as many other species (Nieder and Dehaene, 2009). A number of studies have shown that ANS plays a crucial role not only in the perception of numerosity, but also in understanding symbolic numerals, arithmetic, and mathematics (Malone et al., 2019; Prather, 2019; Sobkow et al., 2020).

Over the past decade, there has been great interest in the role ANS ability plays in higher cognitive processes, such as numerical ability, cognitive reflection, and decision-making. Some studies have demonstrated that high ANS ability is a predictor of good decision-making (Mueller and Brand, 2018; Mueller et al., 2018). The question of whether and how ANS ability relates to higher cognitive functions such as arithmetic, numerical computation, and decision-making is currently receiving much attention, but studies have not yielded consistent results, especially in adults. In developmental studies, it has been shown that ANS ability is closely related to early arithmetic skills, and that ANS ability predicts mathematical achievement in children (Halberda et al., 2008; Mazzocco et al., 2011; Hyde et al., 2014; Libertus et al., 2016; Wang et al., 2016; Cai et al., 2018; Libertus, 2019; Malone et al., 2019, 2021). In line with these arguments, several correlational studies have suggested that ANS ability may be foundational to the acquisition of formal math abilities (Mazzocco et al., 2011; Wang et al., 2016; Malone et al., 2019), and a deficit in the mechanism for representing and processing numerosity has proven to be one of the causes of low ability in symbolic numerical tasks (Butterworth, 2017). These studies suggest that both numerosity comparison and verbal numerical estimation, as well as ANS-number word mapping, may be important for acquisition of math abilities (Libertus et al., 2016). However, evidence from adult studies is not as clear cut as that found in developmental studies; many studies have pointed out that the relationship is inconsistently observed in adults (Yeo et al., 2019; Yeo and Price, 2021). For example, there is evidence that ANS acuity indirectly reflects only certain domains of math achievement in adults (Inglis et al., 2011; Patalano et al., 2015; Jang and Cho, 2016, 2018). Scholars have pointed out that a possible reason for the mixed results might be that different tasks have been used to measure both ANS ability and mathematics achievement (Lindskog et al., 2013; Prather, 2019). The ANS-related tasks used in each study for the purpose of assessing ANS ability have been inconsistent. Typically, there are three types of tasks used to assess ANS ability: numerosity comparison, numerosity estimation, and mental number-line mapping. For example, some studies used the numerosity comparison task in which the participants were presented with a pair of dot arrays, and asked to determine which array contained the larger number of dots (Mueller and Brand, 2018), while others used numerosity estimation tasks in which a number of elements were presented on a screen, and participants had to estimate the approximate numerical values (Yeo et al., 2019). Some studies argue that the acuity of symbolic-number mapping (a measure of approximate numeracy) is a robust predicator of numeracy and decision-making (Sobkow et al., 2019, 2020). To clarify the difference between each of the ANS-related tasks and their relation to arithmetic ability, Guillaume et al. (2016) compared two numerical tasks: numerical comparison and numerical estimation. Their results, which found no relation between the performance of these tasks, demonstrated that numerical comparison and estimation may mediate different cognitive mechanisms (Guillaume et al., 2016). They also tested the relationship between each numerosity task and arithmetic competence, and suggested that the performance of numerical comparison does not provide a pure measure of ANS ability. This evidence calls into question the relevance of correlating this measure with numerical ability, such as arithmetic competence, and underscores the importance of gaining a clear understanding of what each task assesses.

The purpose of the present study was to examine whether and how different ANS tasks relate to cognitive ability, which is highly related to numerical ability in adults in the general population. Specifically, we conducted an online experiment with adults, using three types of ANS-related tasks to assess ANS ability (i.e., numerosity comparison, numerosity estimation, and proportion estimation), and two types of cognitive ability tasks that might be related to ANS ability: numeracy and cognitive reflection tests (CRT). We tested the correlation among the tasks, and examined the relationship between the three types of ANS-related tasks. In addition to the two conventional ANS-related tasks, numerosity comparison and numerosity estimation, we introduced a proportion estimation task. Proportion estimation falls within the framework of perceived numerosity and probability judgments (Varey et al., 1990; Hollands and Dyre, 2000; Slusser and Barth, 2017). Although proportion estimation has not been used to assess ANS ability, we consider it to be an effective measure for assessing ANS ability as estimating proportion requires numerosity perception and the ability to map symbolic numerals. To assess numeracy, we used the Rasch-based numeracy scale (Weller et al., 2013). To assess cognitive reflection, we used CRT (Frederick, 2005; Toplak et al., 2014).

The concept of numeracy is typically defined as the ability to understand and process numerical information (Reyna et al., 2009). This includes computational skills such as multiplying, proportional reasoning, metacognitive monitoring, and understanding the gist of relative magnitude. Some research suggest that individual differences in numeracy may have important consequences for decision-making. CRT is also an extensively investigated measure of individual differences in rationality. This test was originally developed within the dual-process framework (Epstein et al., 1996; Evans and Stanovich, 2013), and captures whether people are able to inhibit their first incorrect response and follow it up with an intuitive and correct response. This score is also positively correlated with superior decision-making in a variety of decision tasks (Sinayev and Peters, 2015; Juanchich et al., 2016). As CRT items consist of mathematical tasks, it is suggested that the test largely captures not only cognitive reflection, but also other aspects related to numerical ability (Liberali et al., 2012; Campitelli and Gerrans, 2014; Patalano et al., 2015, 2020).

Based on the findings of previous studies, we made three predictions: First, no relation would be observed between the performance of numerosity comparison and numerosity estimation. Second, the measures of numerosity comparison and numerosity estimation would independently relate to the numeracy and CRT scores. Third, the proportion estimation measure would relate to the numeracy and CRT scores because both, abilities of numerosity comparison and proportion estimation, were required in performing the task.

## Methods

### Participants

A total of 300 (150 female, 150 male) adults aged 20–39 years participated in the experiment through a Web inquiry company (Cross Marketing Inc.). This age group was chosen as cognitive functions such as spatial visualization, reasoning, and memory and speed are reported to be considerably stable in this age group (Salthouse, 2010). All were native Japanese speakers and residents. Participants were required to use a laptop computer to be eligible to take part in the experiment. There was no regulation on the presentation time for each question and stimulus, and participants could take up the tasks at their own pace. Each numerosity task included these instructions: “There is no need to count dots one by one. Please answer based on your quick impression.” There were no practice trials, and there was no feedback given on the correctness of the choices for any of the tasks.

### Materials and Procedure

All participants performed three ANS-related tasks (numerosity comparison, numerosity estimation, and proportion estimation), Rasch-based numeracy scale, and CRT. Each task is described in the following sections. The questions in numeracy and CRT task are listed in the Supplementary Materials.

#### Rasch-Based Numeracy Scale

The Japanese version of the Rasch-based numeracy scale developed by Weller et al. (2013) was used (Hirota, 2019). The scale consists of eight questions on mathematical expressions and calculation of ratios, and two questions from Frederick’s original CRT (Frederick, 2005). The scale has been used in a wide range of populations, and its usefulness and advantages have been tested (Weller et al., 2013; Peterson and Cheng, 2020). In the present study, participants were asked to answer the questions and record their answers using the numeric keypad on a computer. For each participant, we counted the number of correct answers, and computed the rate of correct response (number of correct responses out of eight) as the numeracy score.

#### Cognitive Reflection Tests

Participants were asked to answer five questions composed of one from Frederick’s original CRT and four from Toplak’s additional CRT (Toplak et al., 2014). The Japanese version of these tests was used (Harada et al., 2018). The number of correct answers was used as the CRT score. Possible scores ranged from 0 to 5 points, with higher scores indicating higher cognitive reflection.

#### Numerosity Comparison Task

**FIGURE 1:**
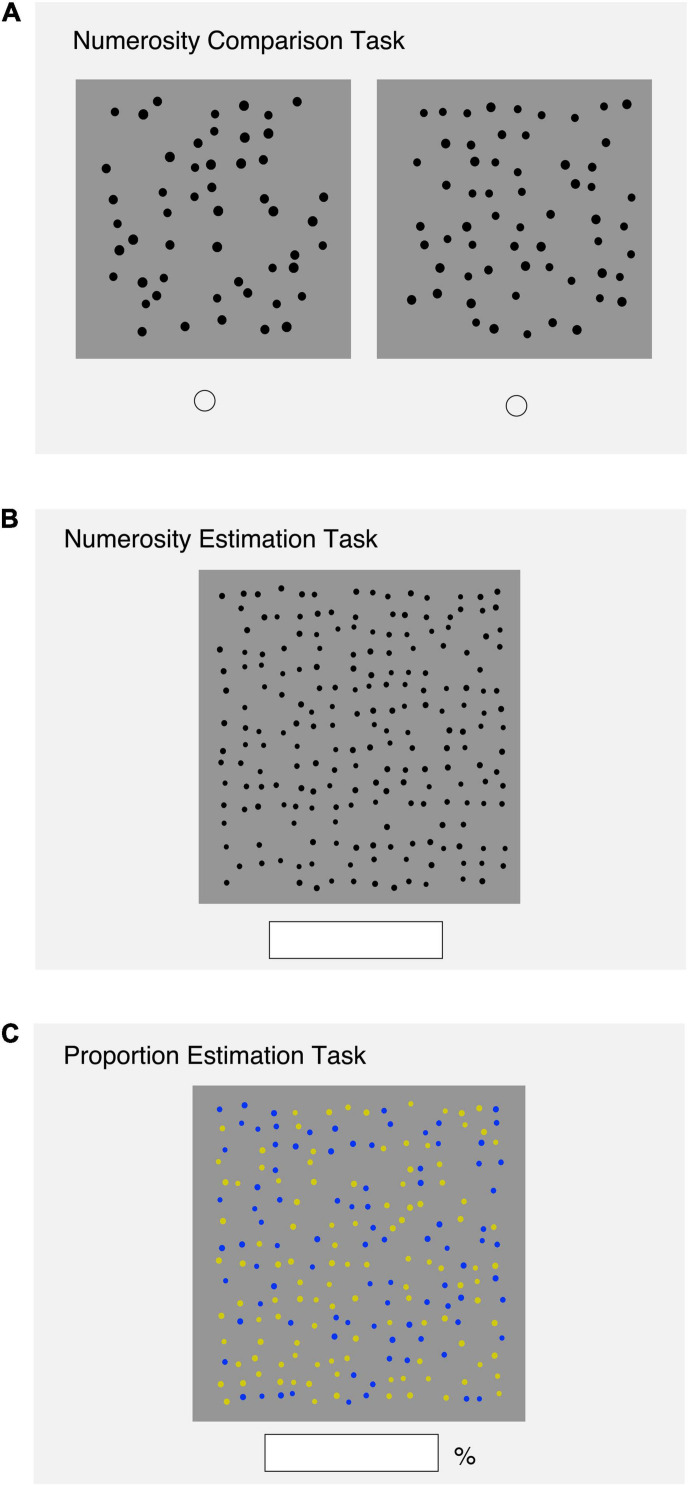
**(A)** An illustration of the numerosity comparison task. **(B)** An illustration of the numerosity estimation task. **(C)** An illustration of the Proportion estimation task.

As shown in Figure 1A, two sets of arrays, a standard array and a comparison array, were presented on the screen. The stimuli consisted of black dots on a light gray background. The diameter of the dots varied from array to array. The average dot size was controlled so that the total area of the dots was not a reliable cue for numerosity. Four standard numbers of stimuli (60, 90, 135, and 202) were used. These were within the range of numerical values presented in the numerosity estimation (40–451) and proportion estimation (15–302) tasks. The ratio of the comparison to the standard values was 0.85–0.9; thus, the sets of arrays were 60/51, 90/77, 135/115, 202/172, 60/54, 90/81, 135/122, and 202/182. These ratios were chosen to ensure the validity of the performance (Lindskog et al., 2013). The presentation order of the trials was randomized within a block. The participants were asked to indicate which array had more dots by clicking on the button below each array. At the beginning of the task, the participants were instructed to judge by the number of dots, and not by other properties of the arrays such as area and density. Each participant performed one trial for each stimuli pair, completing eight trials in total. The correct rate (CR) of each participant was calculated and used as the performance measure.

#### Numerosity Estimation Task

As shown in Figure 1B, the participants saw a set of dot arrays presented on a gray background. Eight sets of dots, 27, 40, 60, 90, 135, 202, 302, and 451, which were logarithmically spaced, were presented in random order. The diameter of the dots varied within and between arrays, and the sizes of the invisible grid also varied so that the occupancy ratio of the dots to grid number ranged from 0.70 to 0.80. Neither the total area of the dots nor the spatial configuration could be a cue to numerosity. Each participant performed one trial in each set; thus, eight trials were performed in total. Participants were instructed to estimate the number of dots, and record their estimates using the numeric keypad as accurately as possible. We computed each participant’s estimation accuracy by calculating the mean absolute error rate (AER) for each stimulus set, and used this value as the performance measure. The slope of the linear regression of the data points for each participant was calculated to assess the bias in numerosity estimation.

#### Proportion Estimation Task

As shown in Figure 1C, a stimulus array was randomly presented at the center of the array. The stimuli consisted of blue and yellow dots on a light gray background. The diameter of the dots in the array varied, ranging from 0.9 to 1.1 times the average size, and the positions of stimuli also varied so that the total area of the dots and the spatial configuration would not be a possible cue to numerosity. There were 10 proportions for each set of dots relative to the total number of dots: approximately 0.05, 0.15, 0.25, 0.35, 0.45, 0.55, 0.65, 0.75, 0.85, and 0.95. Two stimulus set sizes, 202 and 302 total dots, were applied. Specifically, the number of blue and yellow dots that appeared were 11/191, 32/170, 52/150, 72/130, 92/110, 112/90, 132/70, 152/50, 172/30, and 192/10 in set size 202, and 15/287, 45/257, 75/227, 105/197, 135/167, 166/136, 196/106, 226/76, 256/46, and 286/16 in set size 302. Thus, there were 20 conditions in total. Participants were instructed to estimate the percentage of blue dots relative to total dots, and record their estimates using the numeric keypad on a computer as accurately as possible. For each participant, we computed the estimation accuracy by calculating the mean AER in each stimulus set and used the value as the performance measure. The slope of the linear regression of data points for each participant was calculated to assess the bias in proportion estimation.

## Results

### Criteria for Data Exclusion

Data were collected from 300 participants through a Web inquiry company. However, data of questionable reliability were removed according to the following criteria: we excluded untrustworthy responses, such as pressing the button on the same side for all trials in the numerosity comparison task, or entering the same number or a patterned number sequence in the numerosity, proportion estimation, numeracy, and CRT tasks. We also excluded responses that indicated the respondent did not understand the problem (for example, in the proportion estimation task, 191 blue dots out of a total of 202 dots is equivalent to approximately “5%”; however, some participants answered “95%”). Responses that appeared to be typing errors were also excluded, specifically responses that were greater than or equal to ten times higher than the correct answer or/and less than or equal to ten times smaller. We visually scrutinized participants’ responses to assess for any outlying estimates that might have been missed by the trimming procedure described above. Data of 50 participants from all tasks were excluded because we performed within-subject correlation analysis. Then, we calculated the measures of each task; data three standard deviations above or below average were considered outliers and excluded from the analysis. As a result, only the responses of 238 participants were included in the analysis.

### Results of Each Task

#### Rasch-Based Numeracy Scale and Cognitive Reflection Tests

The mean numeracy score of all participants was 56.0% (SD = 25.25). This result is consistent with Weller’s original study, which showed 53.3% (SD = 29.5). The mean CRT task score of all participants was 2.63 (SD = 1.67; *n* = 238) out of 5, equivalent to 51.7% (SD = 33.34). This result is also consistent with previous studies (Harada et al., 2018). The value of Cronback’s Alpha for numeracy and CRT was α = 0.75 and α = 0.70, respectively.

#### Numerosity Comparison Task

**FIGURE 2:**
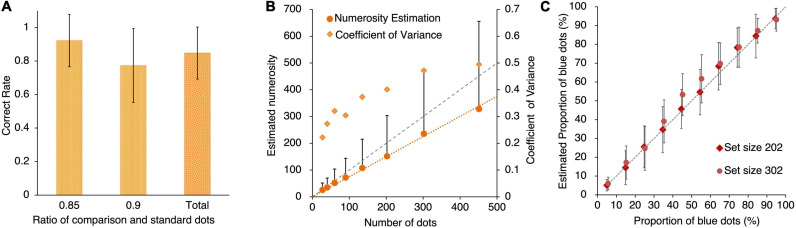
**(A)** Mean correct rate of the numerosity comparison task. Error bars shows the standard deviation. **(B)** Mean estimated numerosity and coefficient of variance (CV) as a function of the number of actual dots in the numerosity estimation task. Error bars shows the standard deviation. **(C)** Mean of estimated proportion of blue dots as a function of actual proportion of blue dots. Error bars shows the standard deviation.

The results of the numerosity comparison task are shown in Figure 2A. The mean correct rates for the ratios of 0.85 and 0.90 were 0.92 (SD = 0.16) and 0.77 (SD = 0.22), respectively. The mean total correct rate was 0.85 (SD = 0.16).

#### Numerosity Estimation Task

Figure 2B shows the average estimated value and variance of each numerosity. The estimated value for each participant’s response was considerably smaller than the actual value. The slope of the regression function for 190 out of the 238 participants was below 1, suggesting that the majority of participants underestimated the objective number of dots. In addition, the coefficient of variance ($C\,V=\frac{S\,D\,o\,f\,e\,s\,t\,i\,m\,a\,t\,i\,o\,n\,a\,c\,r\,o\,s\,s\,p\,a\,r\,t\,i\,c\,i\,p\,a\,n\,t\,s}{m\,e\,a\,n\,o\,f\,e\,s\,t\,i\,m\,a\,t\,i\,o\,n\,a\,c\,r\,o\,s\,s\,p\,a\,r\,t\,i\,c\,i\,p\,a\,n\,t\,s}$) for each numerosity across participants suggests that the variability of estimation increases with the numerosity value. These results are consistent with those of previous studies (Krueger, 1982; Guillaume et al., 2016).

#### Proportion Estimation Task

Figure 2C shows the mean estimated proportion and variance of each proportion. No significant difference was observed in the estimation accuracy between the two set sizes (202 and 302). The slopes for the two sets were below 1.0 (186 out of 238 participants in set size 202, and 190 out of 238 participants in set size 302), demonstrating that the estimated proportions were overestimated when the proportion was smaller, and underestimated when the proportion was larger. These results are consistent with those of previous studies (Varey et al., 1990; Hollands and Dyre, 2000).

The value for Cronbach’s Alpha for a numerosity comparison, numerosity estimation, and proportion estimation was α = 0.43, α = 0.64, and α = 0.58, respectively. It should be noted that the reliability of numerosity tasks were considerably low.

### Results of Correlation Analysis

The Pearson correlation coefficient matrix between the performance measures of the three numerosity tasks, CR from the numerosity comparison task, AERs from the numerosity estimation and proportion estimation tasks, numeracy, and CRT scores, was computed. The mean and standard deviations of all tasks and Pearson’s correlation coefficients between all measures are shown in Table 1.

**TABLE 1:** Bivariate correlations between numerosity discrimination, estimation, proportion estimation, numeracy and CRT.

	***Mean***	***SD***	**Numerosity comparison (CR)**	**Numerosity estimation (AER)**	**Proportion estimation (AER)**	**Numeracy score (CR)**	**CRT score**
Numerosity Comparison (CR)	0.85	0.16	–	−0.17*	−0.20*	0.24**	0.19*
Numerosity Estimation (AER)	0.36	0.14		–	0.30**	−0.25**	−0.37**
Proportion Estimation (AER)	7.89	2.28			–	−0.33**	−0.38**
Numeracy score (CR)	0.56	0.25				–	0.71**
CRT score	2.63	1.67					–

*p < 0.01 and **p < 0.001.

#### Relationships Between Numerosity Measures

**FIGURE 3:**
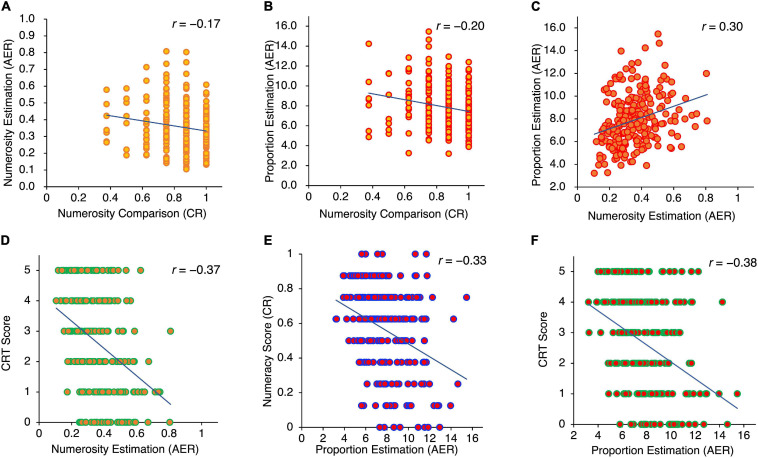
Scatterplots depicting the relation between **(A)** numerosity comparison (CR) and numerosity estimation (AER), **(B)** numerosity comparison (CR) and proportion estimation AER), **(C)** numerosity estimation (AER) and proportion estimation (AER), **(D)** numerosity estimation (AER) and CRT Score, **(E)** proportion estimation (ASE) and numeracy score, and **(F)** proportion estimation (AER) and CRT score.

Figures 3A–C show how the numerosity measures were related to one another. The correlations between the performance of numerosity comparison and numerosity estimation reached a significant level (*r* = −0.17, *p* < 0.01), suggesting a relationship between numerosity comparison and estimation. The results were contrary to our prediction, which was based on previous studies (Guillaume et al., 2016; Prather, 2019). The correlations between the CR in numerosity comparison, AER in numerosity estimation, and AER in proportion estimation reached the significance level with AER in the numerosity estimation (*r* = 0.30, *p* < 0.001) and proportion estimation (*r* = 0.20, *p* < 0.01) tasks.

#### Relationship Between ANS-Related Measures and Numeracy-Related Scores

As expected, the correlation between the numeracy scale and CRT was significant (*r* = 0.71, *p* < 0.001). The relationships between numeracy scores and CR from the numerosity comparison task and AERs from the numerosity estimation and proportion estimation tasks—as well as their respective relationships with the CRT scores—are shown in Figures 3D–F. First, a significant correlation between CR in numerosity comparison and numeracy score (*r* = 0.24, *p* < 0.001), between AER in numerosity estimation and numeracy score (*r* = −0.25, *p* < 0.001), and between AER in proportion estimation and numeracy score (*r* = −0.33, *p* < 0.001) was observed. Participants with higher numerosity comparison measures performed significantly better in the numeracy task than those with lower numerosity comparison measures. Participants with lower AERs in the numerosity estimation and proportion estimation tasks performed significantly better in the numeracy task than those with higher AERs.

A significant correlation between CR from numerosity comparison and CRT score (*r* = 0.19, *p* < 0.001), between AER in numerosity estimation and CRT (*r* = −0.37, *p* < 0.01), and between AER in proportion estimation and CRT (*r* = −0.38, *p* < 0.001) was observed. Participants with higher numerosity comparison measures performed significantly better in CRT relative to those with lower numerosity comparison measures: participants with lower AER in numerosity estimation and proportion estimation performed significantly better in CRT than those with higher AER. In addition, the results showed that the correlation coefficient between CRT score and AER in numerosity estimation [*p* < 0.01, *t*(235) = 2.72] and AER in proportion estimation [*p* < 0.05, *t*(235) = 2.58] was larger than that between CR from numerosity comparison and CRT.

To sum up, the results suggest that the three ANS-related tasks are associated with both numeracy and CRT scores. In particular, it has been suggested that the performance in numerosity and proportion estimation could be a significant predictor of CRT.

## Discussion

The purpose of the present study was to examine whether and how abilities in different ANS-related tasks related to higher cognitive ability associated with numeracy and cognitive reflection in adults. In assessing ANS-related ability, numerosity comparison, numerosity estimation, and proportion estimation tasks were performed. To assess numeracy and cognitive reflection, the Rasch-based numeracy scale and CRT were administered, respectively.

There are three major findings of this study. First, the performance of three ANS-related tasks correlated with each other to suggest that the common numerical ability mediates to carry out these tasks. Contrary to our prediction, there was correlation between performance in the numerosity comparison and numerosity estimation tasks. The findings were in contrast with those of Guillaume et al. (2016) and Prather (2019), who found that performance in the comparison task did not correlate with that in the estimation task, and claimed that numerosity comparison and estimation may mediate different cognitive mechanisms. The findings revealed that there were inconsistencies among the measures assessing ANS ability, as some previous studies had speculated. Why is the relationship between the numerosity comparison and numerosity estimation tasks inconsistent in adult studies? A possible explanation for the absence of a correlation between numerosity comparison and estimation in previous studies could be the sample size. Data from 71 participants were corrected in Guillaume et al. (2016) and data from 30 participants were corrected in Prather (2019). Why has a consistent correlation between numerosity comparison and estimation been observed in developmental studies? It could be attributed to the stimuli number of the estimation task: in development studies, the number of stimuli in numerosity estimation is considerably small, ranging from 5 to 20. In contrast, in adults, it is higher (10–400 or more). With high numerosity, the strategy and cognitive resources for mapping symbolic numerals to perceived numerosity may have large individual differences, as Yeo et al. (2019) claimed. Moreover, although the performance of numerosity comparison is highly associated with that of numerosity estimation in developmental studies, the relationship may change during the process of development.

Second, there was a significant relation between the performance in the numerosity comparison task and Rasch-based numeracy and CRT scores, as well as the performance in numerosity estimation and numeracy and CRT scores. The results support the claim that numerosity processing ability is significantly related to numeracy and cognitive reflection in adults, although the correlation is weak to moderate. The question arises as to how ANS ability relates to cognitive reflection. There are two possible reasons. First, the questions in the cognitive deliberation test used in this experiment included numerical processing skills, such as understanding proportions and calculations. Second, the skill of attending all the items in array, and grasping the approximate numerosity, may share common cognitive ability with cognitive reflection.

Third, it has been proved that the proportion estimation task introduced in this study relates to the performance on numerosity estimation, and the proportion estimation task is more clearly related to CRT score than the numerosity comparison task. The results suggest that proportion estimation could be an effective predicator of numeracy and CRT. As the proportion estimation task requires multiple ANS-related abilities, such as extraction of approximate numerosity and mapping of numerals to perceived proportion, its relation to numeracy and CRT is predicted as well as a single ANS ability.

This study has some limitations that should be considered in future research. First, the experiment was conducted on a large number of 20–39-year-old adults selected online from the general population, and each participant answered the question individually. Therefore, the size of stimuli, presentation time, each participant’s response time, environment, and degree of commitment in performing the tasks were not controlled by the experimenter as this would have involved being in a laboratory. This might cause large individual differences within and between tasks, and raises questions regarding the reliability of the data. It has been suggested that people tend to use economical strategies and minimum cognitive loads in Web experiments, especially when there is no feedback or reward attached. As such, the participants were more likely to have performed the tasks using the least efforts. Second, the reliability indicated by Cronbach’s alpha showed that the reliability of the three numerosity-related tasks was low and that there were differences among the tasks. As the correlation between unreliable items will result in lower values than the actual correlation coefficient, there is a possibility that the correlation between these values may be higher than the results of present studies. More reliable measures to test the numerosity ability for many general participants need to be invented and used for further research. Third, the measures used in proportion estimation and numerosity estimation could be elaborated further. Although previous studies applied these measures, the correct ratio in numerosity comparison and AER in the estimation task may represent a different aspect of numerical ability. To elaborate on the difference between the numerosity comparison and estimation processes, it is necessary to re-conduct the experiment in a laboratory under strictly controlled conditions, and with the appropriate measures for each performance. In addition, it should be noted that although the correlation analysis proves that a relationship exists between ANS-related tasks and numeracy and CRT, it does not prove that higher ANS ability contributes to higher numeracy and cognitive reflection; there is a possibility that numeracy and CRT may influence performance in ANS-related tasks. For example, an understanding of probability and proportion may affect performance in numerosity estimation and/or proportion estimation. The cause-effect relationship between ANS-related ability and numeracy should be examined more concretely. A further study on how each ANS-related ability relates to numeracy and cognitive reflection in adults, and how each ability develops and interacts with one another, should be examined.

## Data Availability Statement

The raw data supporting the conclusions of this article will be made available by the authors, without undue reservation, under reasonable request.

## Ethics Statement

Ethical approval was not provided for this study on human participants because the web survey company used in this study was approved by the participants in advance for ethical issues.

## Author Contributions

MT and SH contributed to conception and design of the study. SH prepared Japanese version of the numeracy test. MT performed the statistical analysis and wrote the first draft of the manuscript. Both authors contributed to manuscript revision, read, and approved the submitted version.

## Conflict of Interest

The authors declare that the research was conducted in the absence of any commercial or financial relationships that could be construed as a potential conflict of interest.

## Publisher’s Note

All claims expressed in this article are solely those of the authors and do not necessarily represent those of their affiliated organizations, or those of the publisher, the editors and the reviewers. Any product that may be evaluated in this article, or claim that may be made by its manufacturer, is not guaranteed or endorsed by the publisher.
