# Supplementary material for: Numerosity Comparison, Estimation and Proportion Estimation Abilities May Predict Numeracy and Cognitive Reflection in Adults
Source: Front Hum Neurosci. 2021 Nov 23;15:762344. doi: 10.3389/fnhum.2021.762344 (PMC8651304; doi:10.3389/fnhum.2021.762344)
Supplement: Supplementary file 1 [file Data_Sheet_1.pdf]

Supplement

Q1	Imagine that we roll a fair, six-sided die 1000 times. Out of 1000 rolls, how many times do you think the die would come up as an even number?
Q2	In the JAMBO LOTTERY, the chances of winning a 1000 yen prize are 1%. What is your best guess about how many people would win a 1000 yen prize if 1000 people each buy a single ticket from JAMBO LOTTERY?
Q3	In the ATARIMASHOW SWEEPSTAKES, the chance of winning a car is 1 in 1000. What percent of tickets of ATARIMASHOW SWEEPSTAKES win a car?
Q4	If the chance of getting a disease is 10%, how many people would be expected to get the disease? Out of 1000?
Q5	If the chance of getting a disease is 20 out of 100, this would be the same as having a _____% chance of getting the disease.
Q6	Suppose you have a close friend who has a lump in her breast and must have a mammography. Of 100 women like her, 10 of them actually have a malignant tumor and 90 of them do not. Of the 10 women who actually have a tumor, the mammography indicates correctly that 9 of them have a tumor and indicates incorrectly that 1 of them does not have a tumor. Of the 90 women who do not have a tumor, the mammography indicates correctly that 81 of them do not have a tumor and indicates incorrectly that 9 of them do have a tumor. The table below summarizes all of this information. Imagine that your friend tests positive (as if she had a tumor), what is the likelihood that she actually has a tumor?

	Tested Positive	Tested Negative	Totals
Actually has a tumor	9	1	10
Does not have a tumor	9	81	90
Totals	18	82	100

Q7	A bat and a ball cost 1,100 yen in total. The bat costs 1,000 yen more than the ball. How much does the ball cost? [Correct answer 50 yen; intuitive answer 100 yen]
Q8	If it takes 5 machines 5minutes to make 5 widgets, how long would it take 100 machines to make 100 widgets? [Correct answer = 5 minutes; intuitive answer =100 minutes]
Q9	In a lake, there is a patch of lily pads. Every day the patch doubles in size. If it takes 48 days for the patch to cover the entire lake, how long would it take for the patch to cover half of the lake? [Correct answer = 47 days; intuitive answer =24 days]
Q10	If Taro can drink one barrel of water in 6 days, and Hanako can drink one barrel of water in 12 days, how long would it take them to drink one barrel of water together? [correct answer = 4 days; intuitive answer = 9]
Q11	Jiro received both the 15th highest and the 15th lowest mark in the class. How many students are in the class? [correct answer = 29 students; intuitive answer = 30]
Q12	A man buys a pig for 6,000 yen, sells it for 7,000 yen, buys it back for 8,000 yen, and sells it finally for 9,000 yen. How much has he made? [correct answer = 2,000 yen; intuitive answer = 1,000 yen]
Q13	Sabro decided to invest 800,000 yen in the stock market one day early in 2018. Six months after he invested, on July 17, the stocks he had purchased were down 50%. Fortunately for Sabro, from July 17 to October 17, the stocks he had purchased went up 75%. At this point, Sabro has: a. broken even in the stock market, b. is ahead of where he began, c. has lost money [correct answer = c, because the value at this point is 700,000 yen; intuitive response =b].

Note. Q1 - 8 are taken from Rasch-based numeracy scale, Q9 - 13 are list of CRT, among them Q9 from Frederick (2005) original Question and Q10 - 13 are added by Toplak et

al. (2014). The underlined text indicates the parts that have been changed for Japanese. Japanese translations for Q1- 9 were by Hirota (2019), and for Q10-13 were by (Harada et al., 2018).
